# The validation status of blood biomarkers of amyloid and phospho-tau assessed with the 5-phase development framework for AD biomarkers

**DOI:** 10.1007/s00259-021-05253-y

**Published:** 2021-03-06

**Authors:** N. J. Ashton, A. Leuzy, T. K. Karikari, N. Mattsson-Carlgren, A. Dodich, M. Boccardi, J. Corre, A. Drzezga, A. Nordberg, R. Ossenkoppele, H. Zetterberg, K. Blennow, G. B. Frisoni, V. Garibotto, O. Hansson

**Affiliations:** 1 Institute of Neuroscience & Physiology, Department of Psychiatry & Neurochemistry, Sahlgrenska Academy, University of Gothenburg, House V3/SU, SE-431 80 Mölndal, Sweden; 2 Department of Old Age Psychiatry, Institute of Psychiatry, Psychology & Neuroscience, King’s College London, London, UK; 3 Wallenberg Centre for Molecular and Translational Medicine, University of Gothenburg, Gothenburg, Sweden; 4 Clinical Memory Research Unit, Department of Clinical Sciences, Malmö, Lund University, Lund, Sweden; 5 Department of Neurology, Skåne University Hospital, Lund, Sweden; 6 Wallenberg Centre for Molecular Medicine, Lund University, Lund, Sweden; 7 NIMTlab - Neuroimaging and Innovative Molecular Tracers Laboratory, University of Geneva, Geneva, Switzerland; 8 Center for Neurocognitive Rehabilitation (CeRiN), CIMeC, University of Trento, Trento, Italy; 9 German Center for Neurodegenerative Diseases (DZNE), Rostock-Greifswald, Rostock, Germany; 10 LANVIE - Laboratory of Neuroimaging of Aging, University of Geneva, Geneva, Switzerland; 11 Centre National de la Recherche Scientifique, Montpellier, France; 12 Medical Faculty and University Hospital of Cologne, Cologne, Germany; 13 Division of Clinical Geriatrics, Center for Alzheimer Research, Department of Neurobiology, Care Sciences and Society, Karolinska Institutet, Stockholm, Sweden; 14 Theme Aging, Karolinska University Hospital Stockholm, Stockholm, Sweden; 15 Alzheimer Center Amsterdam, Department of Neurology, Amsterdam Neuroscience, Vrije Universiteit Amsterdam, Amsterdam UMC, Amsterdam, The Netherlands; 16 Clinical Neurochemistry Laboratory, Sahlgrenska University Hospital, Gothenburg, Sweden; 17 Department of Neurodegenerative Disease, UCL Queen Square Institute of Neurology, London, UK; 18 UK Dementia Research Institute at UCL, London, UK; 19 Memory Clinic, Geneva University Hospitals, Geneva, Switzerland; 20 Diagnostic Department, University Hospitals of Geneva, Geneva, Switzerland; 21 Memory Clinic, Skåne University Hospital, SE-205 02 Malmö, Sweden

**Keywords:** Alzheimer’s disease, Blood, Strategic roadmap, Aβ42, Aβ40, P-tau

## Abstract

**Purpose:**

The development of blood biomarkers that reflect Alzheimer’s disease (AD) pathophysiology (phosphorylated tau and amyloid-β) has offered potential as scalable tests for dementia differential diagnosis and early detection. In 2019, the Geneva AD Biomarker Roadmap Initiative included blood biomarkers in the systematic validation of AD biomarkers.

**Methods:**

A panel of experts convened in November 2019 at a two-day workshop in Geneva. The level of maturity (fully achieved, partly achieved, preliminary evidence, not achieved, unsuccessful) of blood biomarkers was assessed based on the Biomarker Roadmap methodology and discussed fully during the workshop which also evaluated cerebrospinal fluid (CSF) and positron emission tomography (PET) biomarkers.

**Results:**

Plasma p-tau has shown analytical validity (phase 2 primary aim 1) and first evidence of clinical validity (phase 3 primary aim 1), whereas the maturity level for Aβ remains to be partially achieved. Full and partial achievement has been assigned to p-tau and Aβ, respectively, in their associations to ante-mortem measures (phase 2 secondary aim 2). However, only preliminary evidence exists for the influence of covariates, assay comparison and cut-off criteria.

**Conclusions:**

Despite the relative infancy of blood biomarkers, in comparison to CSF biomarkers, much has already been achieved for phases 1 through 3 – with p-tau having greater success in detecting AD and predicting disease progression. However, sufficient data about the effect of covariates on the biomarker measurement is lacking. No phase 4 (real-world performance) or phase 5 (assessment of impact/cost) aim has been tested, thus not achieved.

## Introduction

The “Biomarker Roadmap” initiative was established in 2017 after adapting an oncology methodological framework [1] for the systematic assessment of biomarker validation in Alzheimer’s disease (AD) [2]. Using this 5-phase framework, previous reviews have already assessed the validation and maturity status of well-consolidated biomarkers [3–8]. The Biomarker Roadmap Initiative framework also included the systematic validation of cerebrospinal fluid (CSF) AD biomarkers [9], with recent updates [10]. However, the 2017 framework did not include the assessment of blood biomarkers since robust evidence of blood biomarkers specific for AD pathophysiology was lacking [11].

A blood biomarker offers the opportunity for a widely accessible triage for the rapid assessment of patients in primary care or the identification of appropriate individuals for recruitment into therapeutic trials. While the concept of a blood biomarker for dementia certainly predates the studies examined in this review [12, 13], large-scale explorative omics have failed to identify robust biomarkers for AD dementia or the underlying pathology [14–18]. Instead, due to the advancement of targeted proteomic technologies and the emergence of well-characterized research cohorts (e.g. Alzheimer’s Disease Neuroimaging Initiative (ADNI) and Swedish BioFINDER), the blood biomarkers assessed today are largely based on those already established in CSF [19].

This review focuses on the recent advancements in amyloid-β (Aβ) and phosphorylated tau (p-tau) as leading blood biomarkers that identify AD and its underlying pathology [20]. Though we recognize that neurofilament light (NfL) and total tau (t-tau) have been widely investigated in the context of AD, they are not considered in this review due to non-disease specificity for AD (NfL) or lack of clear disease association (t-tau). Concentrations of blood (plasma or serum) NfL have been shown to be robustly, albeit moderately, increased in MCI and AD [21–25]. Yet, NfL is a well-established measure of global neuronal injury in many neurodegenerative diseases [26, 27] and acute neurological disorders [28]. Thus, NfL does not have the required specificity to be classified as an “AD biomarker”. Plasma t-tau has also been investigated widely in AD [25, 29, 30], yet these studies have concluded that plasma t-tau, certainly in its current immunoassay format, only shows minor changes with large overlaps with disease controls to have clinical relevance for AD. Recent studies, despite being preliminary, have suggested that associations with AD can be achieved by measuring t-tau using an assay format directed toward the N-terminal region of the tau protein in blood [31, 32].

Since the last Biomarker Roadmap Initiative framework in 2017, which included CSF biomarkers [9], blood biomarkers have made substantial progress. Ultrasensitive immunoassays (p-tau and Aβ), fully automated immunoassays (Aβ) and immunoprecipitation mass spectrometry (IPMS; Aβ) methods have been widely reported in large cohorts, predominately defined by CSF biomarkers or in vivo Aβ positron emission tomography (PET). Therefore, the aim of this work is to begin assessing AD blood biomarkers based on the 5-phase framework Biomarker Roadmap methodology.

## Methods

### Target

As mentioned previously, this study was performed with reference to a model imported from the oncology field [1] and adapted to the landscape of AD biomarkers. This literature review examines the validation status of blood p-tau (p-tau181 and p-tau217) and Aβ (Aβ42 or Aβ ratios) as AD biomarkers, in accordance with the 2020 update [33] of the Biomarker Roadmap [2, 3]. For the purposes of this review, all target populations are discussed; AD, AD dementia, MCI and non-AD neurodegenerative disorders, as defined below. Data on familial AD is not the focus of this effort, and data obtained in autosomal-dominant AD are only considered when data in sporadic AD are not available and are not considered for assessing the biomarker maturity. All studies that included targeted blood measures of p-tau and Aβ (e.g. not explorative proteomics) after 2016 were considered. If publicly available data had been utilized for analysis (e.g. ADNI), only the most appropriate study was included in each phase/aim, thus avoiding repeated data and overinterpretation of findings.

### Glossary

Table 1 denotes the terms for this review.

**Table 1:** Glossary term

Term	Meaning
Alzheimer’s disease	The presence of extracellular Aβ plaques and aggregates of hyperphosphorylated tau in NFTs. These features define AD independently of the clinical expression of cognitive symptoms [34]
AD dementia	The developed and progressive decline in memory and other cognitive functions leading to functional impairment in activities in everyday life. Criteria are defined by the National Institute of Neurological and Communicative Disorders and Stroke and the Alzheimer’s Disease and Related Disorders Association criteria [35]. Due to clinical criteria only, AD dementia cases will have non-AD pathology or mixed AD and other types of pathology [36]
Mild cognitive impairment	This refers to individuals without, or with subtle, functional disability but with an acquired objective cognitive impairment. Representing a clinical syndrome, it encompasses cases progressing to AD (about 40–60%) or non-AD dementia (about 10%–30%; [37, 38] as well cases who are stable during several years (about 30–50%). MCI cases positive for AD biomarkers can be defined as prodromal AD based on research diagnostic criteria [39, 40]
Non-Alzheimer’s disease neurodegenerative disorder	Refers to all neurodegenerative disorders considered in the context of AD differential diagnosis. The term is considered independent of the clinical manifestations of these diseases

### Conceptual framework

The conceptual framework for this review stems from the field of oncology [1] and has been described in detail by Boccardi et al. [2] and updated in 2021 [33]. Here, we summarize the application of this methodological framework to the use of blood AD biomarkers, namely p-tau and Aβ, for diagnostic purposes in routine clinical settings. Specifically, all aims are qualified as “fully achieved”, “partly achieved”, “preliminary evidence” or “not achieved” based on the available evidence.

#### Phase 1

Phase 1 studies investigate the rationale for using blood p-tau and Aβ for the diagnosis of AD and AD dementia.

#### Phase 2

Phase 2 aims to define the ability of blood biomarkers to discriminate patients with AD dementia from cognitively unimpaired (CU) and more importantly from non-AD dementias. Phase 2 also defines the clinical assay employed for biomarker measurement and assesses comparisons between assay formats. This phase also aims at identifying possible differential effects of covariates (e.g. age, gender, apolipoprotein ε4 (*APOE* ε4) status) in patients and CU, which may influence the concentrations levels of these biomarkers independently from the disease pathophysiology.

#### Phase 3

Phase 3 studies assess the ability of AD blood biomarkers to detect prodromal disease, e.g. the ability to predict future development of AD dementia in patients with MCI. Given that the large majority of blood biomarker studies are endophenotyped by CSF or PET Aβ, we can also investigate the association to preclinical disease (cognitively unimpaired but underlying pathology (CU+)). Phase 3 studies aim to define criteria for positivity (AD or Aβ+), to compare the diagnostic performance with other biomarkers and to assess the diagnostic value of combinations of biomarkers with a view to defining biomarker-based algorithms.

#### Phase 4

Phase 4 studies assess the performance of blood biomarkers in representative patient cohorts from primary care or memory clinics. The biomarker should have been used to support a clinical diagnosis to patients who are subsequently treated based on the biomarker in question. Phase 4 assesses the benefit of a blood biomarker in early disease detection, as well as their practical feasibility and associated protocol compliance. Preliminary evidence about costs is an additional aim, in view of dedicated studies in phase 5. Phase 4 has not been yet started and will not be discussed in this review.

#### Phase 5

Phase 5 studies evaluate the impact of diagnosis based on blood biomarker biomarkers on society (e.g. cost-effectiveness relative to clinically meaningful outcomes). Phase 5 has not yet started and will not be discussed in this review.

### Evidence assessment

For each of these phases mentioned above, evidence was searched in the literature by two independent raters (NJA and JC) and subsequently assessed to evaluate whether each aim and sub-aim was *achieved*, *partly achieved*, *preliminarily investigated*, or *not achieved* or *not addressed* (Table 2).

**Table 2:** Assessment criteria for the Biomarker Roadmap

Fully achieved	Available scientific evidence successfully replicated in properly powered and well-designed studies. Methodologically sound and well-powered studies have provided convincing evidence that has been replicated
Partly achieved	The available evidence is not sufficiently replicated, or samples are not adequately powered, or studies are faulted with major methodological limitations
Preliminary evidence	Only preliminary evidence is available
Not achieved	Studies are not yet performed at the time of the review
Unsuccessful	Available scientific evidence shows a failure for the biomarker in achieving the aim. Findings in the subsequent roadmap phases should be interpreted with caution

### Search for and selection of papers

The phase aim and sub-aim-specific PubMed search strings are provided as an online resource https://drive.switch.ch/index.php/s/4reUTSuqNZHyIC8.

## Results

**Fig. 1:**
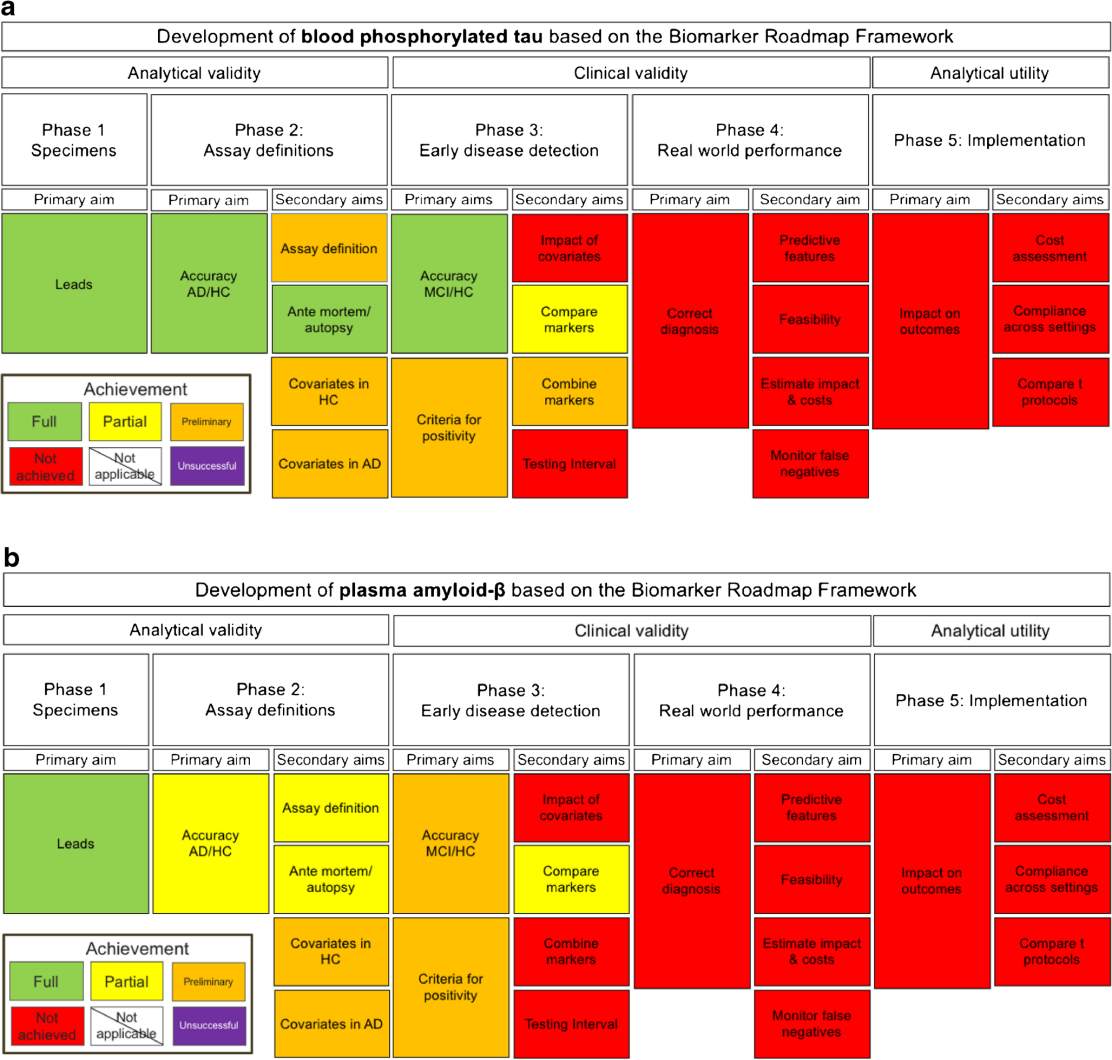
A flowchart illustrating the development of blood biomarkers, p-tau (**a**) and Aβ (**b**) for AD in the framework of Pepe et al. (2001). Abbreviations: *AD*, Alzheimer’s disease; *HC*, healthy controls; *MCI*, mild cognitive impairment

Figure 1A (p-tau) and Fig. 1B (Aβ) provide an overview of the current validation status of blood biomarkers according to our methodological framework.

### Phase 1: preclinical exploratory studies

The aim of phase 1 studies is to identify characteristics unique to AD that might lead to ideas for clinical tests for detecting AD.

#### Phase 1: primary aim

The primary aim of phase 1 is to identify leads for potential biomarkers and to prioritize such identified targets. The idea of a blood biomarker follows the same rationale as for the development of CSF biomarkers for AD, detailed in the 2017 Biomarker Roadmap by Mattsson et al. [9]. The identification of truncated N-terminus Aβ [41] – later Aβ42 in extracellular plaques [42] – tau being identified as a major constituent of neurofibrillary tangles [43] and hyperphosphorylated tau at serine and threonine amino acid residues [44], led to the development of CSF assays for Aβ [45–47], t-tau [48] and p-tau [49].

The decrease in CSF Aβ42 in AD dementia in comparison to healthy controls has been validated in numerous papers [11] and is thought to reflect sequestered of Aβ in extracellular plaques. As a result, lower amounts of Aβ peptides are secreted to the extracellular space, CSF and, potentially, to blood. In agreement, several papers have consistently found a high concordance between CSF Aβ42 and amyloid PET status [50]. The CSF Aβ42/Aβ40 ratio has higher performance to identify AD than CSF Aβ42 as a single biomarker. A multitude of studies also indicate that the CSF Aβ42/Aβ40 ratio shows better concordance with amyloid PET positivity and that the CSF Aβ42/Aβ40 ratio has a clinically relevant diagnostic value [51]. The hypothesized reasoning for this finding is that CSF Aβ40 can serve as a moderator for “total” Aβ levels and that the ratio with CSF Aβ42 normalizes for variation in “total” Aβ production level between individuals. The increase of t-tau and p-tau into the extracellular space is thought to reflect the intensity of neurodegeneration and NFT pathology, respectively [19]. NFTs consist of aggregated tau fragments that are phosphorylated mostly at epitopes in the far mid-region and the C-terminus [19]. P-tau fragments that are poorly retained in the NFTs (e.g. theorine181 and theorine217) are released into CSF and blood as potential biomarkers. However, despite CSF p-tau residues being specific to AD tau pathology, only moderate correlations exist between tau PET, an in vivo measure of NFT pathology [52, 53]. Furthermore, soluble p-tau increase precedes tau PET by a decade [54] and emerge when Aβ pathology is developing [55–58]. Thus, it has been hypothesized that p-tau release into the CSF is indicative of an active process and one that is initiated by cerebral amyloid-β (Aβ) deposition [59]. This is further supported by the fact that MAPT mutations (e.g. R406W) resulting in NFTs consisting of combined 3R and 4R tau (as seen in AD) do exhibit normal CSF p-tau181 and p-tau217 levels level when Aβ plaques are not present [56].

CSF is continuous with the brain extracellular fluid, with a free exchange of molecules between these compartments. However, only a small proportion of brain proteins enter the bloodstream. Blood, while more accessible, is a vastly more challenging matrix to detect brain biomarkers, for several reasons: (1) low abundant brain proteins entering the blood have to be measured in a matrix containing very high levels of peripheral plasma proteins, such as albumin and IgG; and (2) brain proteins are differentially degraded by proteases in blood and metabolized in the liver or cleared by the kidneys, making the interpretation of findings difficult due high variance levels.

Prior to 2016, studies on plasma Aβ failed to replicate the observed decrease in CSF – with no or only minor changes combined with large overlaps in both Aβ42 and Aβ40 between patient and control groups [11, 60]. These disappointing early results have been attributed to the substantial contribution from peripheral tissues to the global pool of plasma Aβ, as evidenced by the poor correlation between plasma and CSF Aβ concentrations [61]. Furthermore, analytical limitations using enzyme-linked immunosorbent assay (ELISA) methods (e.g. epitope masking by hydrophobic Aβ peptides binding to plasma proteins [62]) could be evaded by methodical enhancements. The high analytical sensitivity (< 1 pg/mL) of the single-molecule array (Simoa) allows for predilution of samples that may reduce matrix effects. Subsequently, using this technique for Aβ [63], a significantly lower plasma Aβ42/40 ratio was found in both MCI and AD cases as compared with controls [25, 64]. Furthermore, marginally improved associations were found with CSF Aβ and amyloid PET [64]. Aligned with these immunoassay efforts, immunoprecipitation mass spectrometry (IPMS) studies have attempted to evaluate whether mass spectrometric analysis could provide a more accurate quantification of Aβ peptides in plasma. Pilot studies [65–67] suggested that both Aβ42 and Aβ42/40 were significantly reduced in amyloid PET-positive patients with impressive accuracies (area under curve, AUC) – these studies paved the way for larger studies using mass spectrometric measures of plasma Aβ. These pilot findings using new generation digital immunoassays and enhanced targeted mass spectrometry rejuvenated the interest in plasma Aβ for AD diagnostics. The phase 1 aim is fully achieved for Aβ.

As mentioned above, ultrasensitive immunoassay techniques also allow for the measurement of t-tau in blood [30, 68] but are not diagnostically useful in the context of AD. Evidence from studies in acute hypoxic brain injury demonstrates a biphasic release of tau into the bloodstream that results in a primary peak of plasma t-tau during the first few hours after injury and a secondary, broader peak, that arises a few days after injury and is predictive of neurological outcome [69]. These rapid changes in plasma t-tau concentrations have also been observed in patients with concussion [70] and during anaesthesia [71], which could partially explain the lack of correlation between plasma and CSF t-tau concentrations in AD [29, 72]. Furthermore, t-tau is expressed in peripheral tissues and is also present in saliva with no relationship with AD pathophysiology [73, 74]. An important aspect in developing p-tau residues assays for blood was the discovery that tau fragments in blood consist mainly of N-terminal to mid-region forms [59], which are different from the mid-region targeted by CSF assays [75]. Therefore, p-tau assay development for blood has focused on these species. For instance, Tatebe and colleagues [76] developed a p-tau181 assay which was modified from the Simoa t-tau assay, substituting the detection antibody for a p-tau181-specific monoclonal antibody. This was the first study to report significant increases of plasma p-tau181 in AD dementia and Down syndrome patients, yet still suffered from analytical sensitivity. Mielke and colleagues [77] then described an electrochemiluminescence (ECL) method, developed by Eli Lilly, to measure p-tau181 which also reported highly significant increases in AD but importantly demonstrated a strong relationship with amyloid and tau PET. This relationship with PET – as well as with CSF measures – was later shown to become abnormal at early stages of AD pathogenesis development [78]. A method to quantify plasma and serum p-tau181, more sensitive than the Simoa assay previously described, was then later developed [79]. These studies are the basis for the accumulating evidence for measuring p-tau181 by Simoa [79] and MSD [80, 81] as well as p-tau217 [82] and more recently p-tau231 [83]. The phase 1 aim is fully achieved for p-tau.

### Phase 2: clinical assay development for clinical disease

The general aim of phase 2 studies is to define the ability of blood biomarker assays to discriminate AD dementia patients from CU and non-AD dementias.

#### Phase 2: primary aim

The primary aim of phase 2 is to estimate the true-positive rate and false-positive rate or receiver operating characteristics curve for the assays and to assess their ability to distinguish subjects with and without AD.

Significantly lower plasma Aβ42/40 ratios have been reported in both MCI and AD as compared with CU [64, 84–88]. However, the primary outcome of these plasma Aβ studies has predominately been to detect Aβ positivity. The Aβ42/40 ratio in plasma is reduced by 14–20% in amyloid PET-positive individuals [64, 66, 86, 87]. In comparison, a 50% reduction is observed in CSF for Aβ42/40 [11]. As such, plasma Aβ shows a greater overlap between amyloid PET-positive and amyloid PET-negative individuals. Yet, common findings are emerging between Aβ IPMS studies and the fully automated Aβ immunoassay (Elecsys) [85]. In addition to Aβ42/40, MS-based studies also suggest that a ratio of an APP fragment (APP669-711) to Aβ42 in plasma identifies Aβ-positive individuals [87]. Plasma APP669-711/Aβ42 ratio was 20–40% higher in Aβ + individuals than in Aβ − individuals, which gave high sensitivity and specificity for predicting Aβ status in AD and MCI patients, as well as in CU (91% and 87%, respectively) [87]. Studies comparing AD and non-AD dementias using plasma Aβ assays are few. Janelidze et al. [80] demonstrated that plasma Aβ42/40 was significantly reduced in MCI Aβ + when compared to MCI Aβ − patients (which infers a non-AD pathology). Furthermore, Palmqvist et al. [82] also demonstrated that the median plasma Aβ42/40 ratios were lower in neuropathologically confirmed AD when compared to non-AD neurodegenerative diseases. The same study also demonstrates a reduction of plasma Aβ42/40 of AD dementia compared to non-AD neurodegenerative diseases in the BioFINDER cohort but was significantly inferior to other biomarkers analysed. Lin et al. [89] measured plasma Aβ42 concentrations in healthy individuals and non-AD dementias. Of all the groups included in the study, dementia with Lewy bodies (DLB) had the lowest plasma Aβ42 concentrations, although the reduction was not statistically significant. Individuals with frontotemporal dementia (FTD) had significantly higher blood Aβ42 concentrations compared to all other groups. This finding is particularly interesting given the low frequency of Aβ retention in the brains of individuals with FTD [90]. There is a large agreement in the change of plasma Aβ42/Aβ40 (or variations of such) in AD patients as compared CU Aβ − controls, which are seemingly more promising for mass spectrometric methodologies than immunoassays. However, there are limited dedicated studies on the performance of Aβ assays in non-AD dementias. The primary aim of phase 2 is thus only partially achieved for Aβ.

A breakthrough discovery in the mid-1990s demonstrated that AD patients had increased levels of CSF p-tau, which was not found in other neurological diseases [48]. Later studies have since confirmed that CSF p-tau181 can be used to differentiate AD from other dementias, such as frontotemporal dementia (FTD) and dementia with Lewy bodies [91–93]. Recently, five studies have shown that CSF p-tau217 perform somewhat better than CSF p-tau181 when differentiating AD from non-AD diseases [92–96]. These findings have now been largely replicated using blood p-tau. P-tau181 has been quantified using both the Simoa [79, 97, 98] and MSD platforms [80, 81] which utilizes different antibodies. Janelidize et al. described high accuracy (AUC = 0.93, sensitivity = 0.92, specificity = 0.87) in detecting AD from non-AD neurodegenerative disorders [80] which was comparable to CSF measures of Aβ and t-tau and only slightly inferior to CSF p-tau181 and tau PET. Similarly, Karikari et al. [79] demonstrated a complete separation between AD and FTD patients. In the replication cohort, the high accuracies also observed in distinguishing between AD and non-AD, however, demonstrated more variability, which was dependant on the non-AD diagnosis (vascular dementia, AUC = 0.92; atypical parkinsonian disorders, AUC = 0.89; behavioural FTD and Parkinson disease, AUC < 0.85). Furthermore, high accuracy in differentiating AD from FTLD patients has been observed in two other studies using serum [97] or plasma [81] p-tau181. A recent publication showed that plasma p-tau217 discriminates clinically diagnosed AD from non-AD dementias with the same accuracy as CSF p-tau217 and tau PET imaging (all with AUCs > 0.95), and the levels in the plasma of p-tau217 are 5–7-fold increased in AD dementia compared to other neurodegenerative diseases [82]. The usefulness of plasma p-tau217 was supported by a recent mass spectrometry-based study [99].

Plasma p-tau181 and p-tau217 have been validated in longitudinal studies using ante-mortem collected blood samples and post-mortem histopathology. In the paper by Palmqvist et al. [82], plasma p-tau217 could detect cases with a high likelihood of AD according to the NIA-Regan criteria with an AUC of 0.98 [82]. Similarly, high performance was found in a study using plasma p-tau181 [98]. The primary aim of phase 2 is fully achieved for p-tau.

#### Phase 2: secondary aim 1

The secondary aim 1 of phase 2 addresses the optimization of the operating procedures and assessment of the reproducibility of the assay within and between laboratories. A large amount of work has been published on the preanalytical factors of CSF biomarkers. These advancements have been highlighted as the most important achievements in the updated Biomarker Roadmap for CSF biomarkers [10] – however, at this time, a limited amount of published data on assay comparisons and preanalytical parameters have been reported for blood biomarkers. Yet, a general guideline for blood biomarkers research has been published [100].

The first effort in comparing analytical methods for Aβ has been achieved by the GBSC; as presented at Alzheimer’s Association International Conference 2019, identical aliquots of 81 EDTA plasma samples were sent to 11 laboratories and analysed using standard immunoassays (ELISA, Simoa and Elecsys) or immunoprecipitation mass spectrometry (IPMS) methods. Correlations of Aβ42 concentrations were absent or weak (standard immunoassays) or moderately strong (IPMS methods), while Aβ40 offered more consistency. Clearly, additional assay harmonization and standardization work are required. A recent report [101] has investigated the preanalytical variables of the Elecsys Aβ42 and Aβ40 quantification which showed that these biomarkers were not affected by up to three freeze/thaw cycles, five tube transfers or the size of a collection tube. The same study demonstrated a small diurnal variability for plasma Aβ42 and Aβ40 but no effect was observed for the Aβ42/Aβ40 ratio. Compared to EDTA plasma, an increase and decrease of Aβ biomarkers was observed for lithium heparin and sodium citrate, respectively. Finally, Aβ was stable up to 6 h at +4 °C but only 1 h at room temperature. Partial evidence exists for phase 2 secondary 1 for Aβ.

To date, no intra-laboratory comparison of the most widely reported p-tau assays from the University of Gothenburg [79] and Eli Lilly [77, 82] has been published. The methodology for the Simoa assay has been fully reported [79] and has been successfully transferred to a commercially validated assay by Quanterix, offering an indirect verification of the method. We are also aware that p-tau values in serum are lower than for plasma [79] and that multiple freeze-thaws may effect p-tau values (Ashton et al., Alzheimers Dement (Amst), accepted). No reports on how p-tau can be affected by operator-influenced preanalytical variables have been published but is an ongoing aim by the Global Biomarker Standardization Consortium (GBSC). There is only preliminary evidence for phase 2 secondary 1 for p-tau.

#### Phase 2: secondary aim 2

The secondary aim 2 of phase 2 is to determine the relationship between biomarker tissue measurements made on brain tissue and the biomarker measurements made on the noninvasive clinical specimen (e.g. blood).

Investigations examining the relationship between plasma Aβ and neuropathologically confirmed cases are limited. As previously mentioned, Palmqvist et al. [82] demonstrated marginal changes between neuropathologically confirmed AD and non-AD cases using Aβ assays. The lack of studies comparing these modalities may be due to the unconvincing correlations between plasma Aβ and cerebral Aβ [64, 85, 102], the large volumes (250–1000uL) required for IPMS studies [86, 87], propriety immunoassay technologies [85] or simply that recent evidence demonstrates that plasma p-tau demonstrates a stronger relationship with in vivo Aβ than plasma Aβ itself [79–81, 85]. Nonetheless, plasma Aβ has shown consistent utility in separating Aβ + and Aβ − individuals determined by PET. For IPMS studies, the AUC for Aβ42 ranges from 72–87% [67, 87], between 80–97% for Aβ42/Aβ40 [66, 67, 86, 87] and between 82–97% for the APP669-711/Aβ42 [67, 87]. In contrast, accuracies of between 60–64% and 62–68% have been reported for the commercially available Simoa assays [25, 64, 103], although higher AUCs were achieved for a modified version of the Simoa assay which utilized differing antibodies [102]. Plasma Aβ42/Aβ40 measured by the fully automated Elecsys assay predicts Aβ status with an accuracy > 80% [85]. No in vivo Aβ prediction has been reported for IMR [68], MSD [104] or plasma exosome-bound Aβ42 (APEX) [105]. Interestingly, plasma biomarkers (both Aβ and p-tau) have already been shown to significantly change approximately at the same point as the corresponding CSF biomarkers [78]. The secondary aim of phase 2 is partially achieved for Aβ.

To date, there have been five studies that have addressed the relationship between plasma p-tau measurements and post-mortem data. All studies confirmed that plasma p-tau could separate AD pathology from non-AD pathology with high accuracy [80–83, 98]. It was reported that plasma p-tau217 had a high accuracy in predicting AD pathology from non-AD pathology and demonstrated a strong relationship between p-tau217 and NFT density score in AD – which was not observed for non-AD [82]. Lantero Rodriguez et al. [98] demonstrated that plasma p-tau181 predicts AD pathology at least 8 years prior to neuropathological confirmation and increased with severity of Braak staging. This study also confirmed that longitudinal increases are attributed to NFT pathology which plateaus at end-stage disease. As previously mentioned, several studies have now emerged that plasma p-tau correlates strongly with Aβ and tau pathology in AD [77–82, 106, 107] but plasma p-tau and CSF p-tau only correlate in the presence of Aβ pathology. In support of this, plasma p-tau is seen to be increased in Braak 0 patients (e.g. tau PET-negative) if Aβ is already present [79]. Therefore, plasma p-tau is sensitive to both Aβ and tau pathologies but begins to increase in response to or concurrently with Aβ. All findings demonstrated by CSF p-tau [9, 10] have been replicated in blood, corroborating that p-tau is a robust blood biomarker for AD pathology. The secondary aim of phase 2 is fully achieved for p-tau.

#### Phase 2: secondary aim 3 and secondary aim 4

The secondary aims 3 and 4 of phase 2 are to assess covariates (e.g. sex, age) associated with biomarker status or level in CU (secondary aim 3) and disease (secondary aim 4) subjects. If there is an effect on the biomarker, these covariates may be considered when defining thresholds for positivity in each concerned subpopulation. The effect of demographic factors on blood biomarkers in CU participants or disease patients has not been studied in detail but has been reported as part of the description the biomarker performance in various studies. Increasing age and *APOE* ε4 carriership are associated with AD [108]; as such, blood AD-related biomarkers are more commonly affected in disease groups.

No sex differences in CSF Aβ42 concentrations have been found for any disease stage or *APOE* genotype [109], and therefore we may expect the same relationship to exist for plasma Aβ. Schlinder et al. [86], however, described that plasma Aβ42/40 decreased significantly in males and *APOE* ε4 carriers. Palmqvist et al. [85] reported no age effect of Aβ levels in all diagnostic categories and also demonstrated that the prediction of Aβ positivity did not differ in younger (< 72 years) as compared to older (> 73 years). In contrast, older age was assocaited to a decrease of plasma Aβ42/40 and was a significant contributor to the prediction of Aβ + in IPMS Aβ studies [86]. This finding was also found in an earlier report [110], but how these disparities are influenced by assay differences is not known. For Aβ, preliminary evidence exists for secondary aims 3 and 4 in phase 2.

In some p-tau studies, demographic factors were included in a linear regression model, suggesting an incidental effect on the biomarker performance, but were not individually reported. Karikari et al. [79] and Ashton et al. [83] presented data from young CU individuals (< 40 years) and demonstrated a significant reduction as compared to CU elderly adults without amyloid pathology (> 65 years) in p-tau181 and p-tau231, respectively. Further, Tatebe et al. [76] demonstrated a positive correlation between age and p-tau181 in individuals with Down syndrome, with weak correlations reported by Lantero Rodriguez et al. (*r* = 0.25–0.30) [98] and no relationship reported by Thijssen et al. [81]. This does suggest that age may have a minor influence on the concentrations of p-tau, but to what degree this is independent of undetectable accumulating cerebral Aβ is unclear. Plasma p-tau217 has been studied in autosomal-dominant AD [82], where a positive correlation with age was only observed in mutation carriers. In aged-matched non-carriers, no correlation between age and p-tau was observed which is in line with CSF p-tau associations with age [111]. This highly suggests that a correlation between age and p-tau is dependent on Aβ pathology. Thus far, no significant reports of sex differences have been reported [81, 98]. For p-tau, preliminary evidence exists for secondary aims 3 and 4 in phase 2.

### Phase 3: longitudinal repository studies

The general aim of phase 3 studies is to define the ability of the biomarker to detect the disease in its early phase, e.g. MCI in the context of the Biomarker Roadmap.

#### Phase 3: primary aim 1

The primary aim 1 of phase 3 is to evaluate the capacity of the biomarker to predict the subsequent development of AD dementia in patients with mild cognitive impairment.

While minimal data is available for the future risk of dementia with Aβ measures, Janelidze et al. [80] have demonstrated no association of Aβ42/40 and increased risk (HR = 0.81 95% CI = 0.64–1.02). Using longitudinal cognition (MMSE) and conversion to AD dementia as outcomes in MCI patients, Cullen et al. [112] showed a model combining plasma p-tau181 and NfL, but not Aβ42/Aβ40 (Elecsys assay), had the best prognosis performance of all models. Moreover, this finding held when performing a sensitivity analysis using Aβ42/Aβ40 from a mass spectrometry assay (Araclon Biotech Ltd). Verbeck et al. [102], while they did not perform a future risk analysis, showed that Aβ were less associated to cognitive domains than plasma GFAp and NfL. Conversely, using IPMS Aβ measures, individuals with negative amyloid PET scans at baseline but a positive plasma Aβ42/Aβ40 had a 15-fold greater risk of conversion to amyloid PET positivity [86], which could be valuable for therapeutic trial recruitment.

Plasma p-tau181 and p-tau217 are increased at the MCI stage, if Aβ is present [77, 79, 80, 82, 106, 107]. The increase from Aβ + MCI to AD dementia is much less pronounced, however, and in most cases is nonsignificant. However, a larger increase from Aβ + MCI to AD dementia is observed for p-tau217 [82]. If Aβ is not determined, the differences between CU and MCI remain significant but with larger overlaps and the interpretation becomes much less clear [25, 79, 98, 107]. It also must be noted that plasma p-tau significantly increases in preclinical AD (e.g. Aβ + CU). Plasma p-tau181 has been shown to have an accuracy of between 70 and 82% for predicting Aβ status at the CU stage [75, 79, 80, 82] whereas plasma p-tau217 is significantly higher (> 90%) [82]. Plasma p-tau at baseline is a strong predictor of progression to cognitive decline which is seen to be comparable to CSF p-tau [79, 80, 107]. In the study by Janelidze et al., [80] it was found that individuals who had abnormal baseline levels of p-tau181 had a substantially increased risk of developing AD dementia in the future (HR = 10.9, 95% CI = 5.0–24.0). In a similar manner, using the ADNI data resource [113], higher plasma p-tau181 in MCI patients (HR = 22.75, 95% CI = 9.90–52.3) and Aβ + CU individuals (HR = 3.25, 95% CI = 1.12–9.40) had a greater risk of developing AD dementia over a 48-month period. The hazard ratios (HR) observed for plasma where similar for CSF results from the same patients (MCI, HR = 37.1, 95% CI = 15.0–91.8; HR Aβ + CU = 5.4, 95% CI = 1.8–16.3) [79]. In a follow-up study, we have found that plasma p-tau181, together with plasma NfL, can be used for individualized risk prediction of conversion to AD dementia at 4 years of follow-up in patients with MCI in both the BioFINDER and ADNI studies [112]. To facilitate the use of these plasma-based models, the markers were incorporated into an online tool that can be used for individualized prognosis in MCI (www.predictprogression.com). Phase 3 primary aim 1 is fully achieved for p-tau.

#### Phase 3: primary aim 2

The primary aim 2 of phase 3 is to define criteria for a positive biomarker test in preparation for phase 4. As stated by Leuzy et al. [10], a variety of statistical approaches have been proposed to dichotomize continuous AD biomarkers as normal or abnormal. In clinical chemistry, biomarker cut-offs are commonly defined as the 95% confidence interval in people without disease – as proposed for plasma NfL [27]. So far, a small number of studies have reported concentration cut-offs based on the maximum of accuracy for the target or choosing a cut-point that yields a predefined level of sensitivity or specificity.

Elecsys Aβ42/Aβ40 cut-off of 0.065 was validated in an independent sample with similar AUCs [85]. Further, estimates from a linear regression model that incorporated Aβ42 and Aβ40 as separate measures resulted in higher AUCs in the validated cohort (AUC = 0.86). In applying the regression model of Aβ42, Aβ40 and *APOE*, it was shown that PET costs could be reduced by approximately 1/3 in a typical trial design for Aβ therapeutics. In IPMS methods, a plasma Aβ42/Aβ40 cut-off of < 0.1218 was reported for Aβ PET-positive individuals, with a positive predictive value of 0.88 (95% CI 0.75–0.96) and a negative predictive value of 0.76 (95% CI 0.67–0.83) [86]. An impressive concordance of plasma and CSF Aβ42/Aβ40 (84%) was reported if these cut-offs were applied in an independent sample. In an earlier discovery study, employing the same technique, an optimal cut-off value of < 0.1243 of the plasma Aβ42/Aβ40 concentration ratio was reported [66]. This highlights the robust measurement of Aβ42 and Aβ40 across studies, using both automated immunoassay and mass spectrometric techniques. Thus, efforts have been made to report cut-off for various Aβ assays; however, these cut-offs have not been tested fully by independent laboratories. Preliminary evidence exists for Aβ in phase 3 primary aim 2.

Karikari et al. [79] estimated a cut-off of 15.9 pg/ml for p-tau181 from a small discovery cohort (*n* = 37) which performed favourably in the TRIAD (*n* = 226) and BioFINDER (*n* = 763) validation cohorts. This cut-off was also applicable to independent cohorts in subsequent publications [97, 98, 114]. In the ADNI multicenter study [80], which utilized the same Simoa platform, a cut-off of 17.7 pg/ml for AD diagnosis (14.5 pg/mL for Aβ positivity) was reported which is comparable to the previous cut-off generated from a small sample set. This analysis in ADNI indeed demonstrated a substantial estimated cost-saving if applied in a therapeutic trial recruitment strategy for preclinical AD [107]. These findings support the robustness of the Simoa assay and plasma p-tau181 as a biomarker for routine use across clinical settings and laboratories. It is important, however, to understand that these cut-offs cannot be translated to other p-tau assay platforms. For example, on the MSD platform, Janelidze et al. [80] reported a cut-off of 1.81 pg/mL, for differentiation for a future conversation to AD dementia, while Thijsen et al. [81] reported 8.4 pg/mL for Aβ positivity. Although these are not the same diagnostic parameters as described for Simoa assay studies above, it demonstrates the discrepancies in relative biomarker concentrations reported across these assays and potentially, across cohorts. Preliminary evidence exists for p-tau in phase 3 primary aim 2.

#### Phase 3: secondary aim 1

The secondary aim 1 of phase 3 is to explore the impact of covariates on the discriminatory abilities of the biomarker in the early disease phase. This aim is not achieved for either p-tau or Aβ. Using CSF biomarkers as reference, no adjustment for age, sex or *APOE* ε4 allele has been recommended [10] – but this cannot be confirmed for blood biomarkers as of yet.

#### Phase 3: secondary aim 2

The secondary aim 2 of phase 3 is to compare the different biomarkers available in order to select the most promising ones.

In p-tau studies where other biomarkers have been compared, p-tau alone was deemed the best predictor of AD [25, 79, 80, 82]. Yet, the combination of Aβ42/Aβ40 with p-tau may have a slight improvement in predicting Aβ status [80]. Ratios of p-tau with plasma Aβ42 or t-tau also do not improve diagnostic accuracy [82].

As stated earlier, in most instances, Aβ42/Aβ40 outperforms Aβ42 alone, and Nakamura et al. [87] described the APP669-711/Aβ42 ratio being superior to both. In other IPMS studies, Schindler et al. described the superiority of plasma Aβ42/Aβ40 combined with age and *APOE* ε4 status [86]. Employing the automated Elecsys platform, Palmqvist et al. [85] demonstrated the improvement of plasma Aβ42/Aβ40 from 80 to 86% accuracy if combined with plasma t-tau, plasma NfL and *APOE* status. A recent study by Verberk et al. [102] showed that the combination of plasma Aβ42/Aβ40 and GFAp provides a superior prediction of any biomarker alone – however, p-tau was not included in this study. The correlations between plasma and CSF levels of Aβ42 and Aβ40 were poor in these studies, further supporting that a peripheral production of Aβ is a confounder. Phase 3 secondary aim 2 is partially achieved for plasma Aβ and p-tau but the superior assays in both modalities (e.g. IPMS Aβ and p-tau) have yet to be combined in disease cohorts.

#### Phase 3: secondary aim 3

The secondary aim 3 of phase 3 is to develop algorithms for the biomarker-based diagnosis of MCI in preparation of phase 4. Several studies have explored whether the detection of prodromal AD can be improved by combining CSF AD biomarkers with neuroimaging [10]. However, the context of use and main advantage of blood biomarker is its wide-scale accessibility. It is unlikely that a blood biomarker will outperform CSF and will be better placed as a triage tool for further investigations. Therefore, it is counterintuitive to combine blood with CSF/PET in a prediction model. It is conceivable to combine cognitive tests or simple MRI measures with blood biomarkers, and this would relate to a different context of use than a diagnosis in memory clinics, such as screening in the population or case finding in primary care contexts. Combining plasma Aβ42/Aβ40, p-tau181 and NfL in patients with MCI from BioFINDER and ADNI, Cullen et al. [57] compared the prognostic ability of plasma biomarkers to the same biomarkers measured in CSF, as well as to a more basic model comprising age, sex, education and baseline MMSE. Using cognitive decline and progression to AD dementia over 4 years as outcomes, the authors calculated risk probabilities at the individual patient level using linear regression combined with internal and external validation analyses. Plasma-based models were, overall, non-inferior or better than CSF-based models and superior to the basic model. As mentioned above, these prediction models were also incorporated into an online tool (www.predictprogression.com) providing individualized risk estimates in MCI. Preliminary evidence exists for p-tau for phase 3 secondary aim 3, while this aim remains is not achieved for Aβ.

#### Phase 3: secondary aim 4

The secondary aim 4 of phase 3 is to determine a biomarker testing interval for phase 4 if repeated testing is of interest. Longitudinal measurements of plasma p-tau have revealed low intra-individual variability, which could be of potential benefit in disease-modifying trials seeking a measurable response to a therapeutic target [25, 98, 106, 113]. However, the stability of p-tau has not been common in all studies, namely in the longitudinal evaluation of familial AD [114], which may reflect different preanalytical protocols or a difference in how p-tau is expressed in familial AD. This aim is not achieved.

## Discussion

In this review, we aimed to assess the maturity of blood biomarkers for AD, p-tau and Aβ, according to an oncology-based validation framework adapted for use with AD biomarkers [1, 3]. The validation status for CSF was included in 2017 [9] and has been updated in this edition [10].

Plasma p-tau (p-tau181 and p-tau217) and Aβ (Aβ42/Aβ ratios) were selected for their direct application for AD (phase 1). However, while CSF t-tau is considered in the CSF framework, plasma t-tau was omitted due to the previously published evidence demonstrating limited validity as an AD biomarker [25, 29, 30, 115]. Furthermore, while plasma NfL is the most published blood biomarker related to AD, it was also not considered due to its global association to neurodegeneration [26, 27] and neurological injury [28, 116] (e.g. nonspecificity for AD). Furthermore, the recent development of GFAp as an AD biomarker [25, 102, 107] has not been considered.

There is sufficient evidence that plasma p-tau fulfils the criteria for full achievement in identifying AD (phase 2; primary aim 1), early disease (phase 3; primary aim 1) and has tissue-biofluid association (phase 2; secondary aim 2). The performance of p-tau in these categories is highly impressive, with the prediction of diagnostic groups and dichotomized pathology status comparable to in vivo CSF and PET measures. Furthermore, baseline increases of p-tau biomarkers are strong predictors of disease progression, performing similarly to CSF p-tau181. To date, there have been twelve independent data reports on plasma p-tau181 and two on plasma p-tau217. Yet, the limited evidence available may suggest some superiority of plasma p-tau217 over plasma p-tau181 [82], which is in line with CSF comparisons of the same biomarkers [92, 93, 117]. However, a biomarker comparison using the same detector antibody in sandwich immunoassay format has yet to be performed, and therefore it cannot be determined if the observed superiority is either assay or epitope dependent. It must be noted that the advantage of p-tau217 over p-tau181 has been demonstrated using mass spectrometry [99]. One recent report examines p-tau231 in blood [83] and suggests that while there is no diagnostic advantage over p-tau181, p-tau231 increases early in preclinical disease. Plasma Aβ42/Aβ40, which is superior to plasma Aβ42, only has achieved partial fulfilment for the same criteria fully achieved by p-tau. Although impressive accuracies have been described for IPMS and Elecsys methods, the complexity and availability of platforms have limited their investigation in larger and multiple independent cohorts.

In summary, p-tau demonstrates two clear advantages over Aβ, which have immediate clinical application. Firstly, p-tau has been robustly shown to identify AD in cases with dementia – with non-AD dementias having levels similar to PET Aβ − controls. These findings have already been widely replicated and validated in numerous neuropathological cohorts. This opens the possibility of routinely using plasma p-tau to improve the confidence in an AD diagnosis in primary care and administering symptomatic treatment (e.g. acetylcholinesterase inhibitors or memantine). Importantly, a “negative” plasma p-tau measure, in the presence of cognitive deficit, would highlight the requirement of further examination of suspected non-AD dementia (e.g. structural MRI, DAT scans, FDG-PET) without delay. Secondly, plasma p-tau (or p-tau-based models) highlights the future risk of dementia to the same degree as CSF biomarkers. This would be a rapid indication of those at greater risk, informing on patient management and administering disease-modifying drugs once available. At this time, Aβ measures have not shown to have these clinical capabilities; however, plasma Aβ may have utility in highlighting the risk of future Aβ deposition and contribute to an accurate prediction model of preclinical AD.

While p-tau, and to a lesser degree Aβ, has rapidly achieved success, they are lacking in assay comparisons (phase 2; secondary aim 1). Cut-offs derived for immunoassay and IPMS measures of p-tau and Aβ have achieved replication (or similar cut-off values) in validation studies but across laboratory comparisons are currently not available. Furthermore, detailed descriptions of the impact of covariates in healthy ageing (phase 2; secondary aim 3) and disease (phase 2; secondary aim 4) are lacking. This is where the biggest improvement has been achieved for CSF biomarkers [10]. The optimization of operating procedures and assay reproducibility is now fully achieved with a protocol for the handling of CSF AD biomarkers [118]. Furthermore, phase 3 is also fully complete for CSF biomarkers, which has allowed for reported criteria on positivity and preliminary evidence for phase 4 which aims to support the feasibility of the widespread use of CSF AD biomarkers. Though plasma biomarkers have yet to achieve the same feat, this is likely to be fully investigated soon given the relative ease and acceptance of venipuncture. A key and often overlooked aspect to fluid biomarker assessment, which remains an issue in CSF biomarkers [10], is the assessment of AD blood biomarkers in more diverse ethnic groups. Recently, however, some progress has been achieved in this area for plasma t-tau, plasma NfL and plasma GFAp [119].

In order to understand AD biomarkers in blood, it is important to understand what an immunoassay or mass spectrometric technique is capturing and what it represents. CSF p-tau181 (and other p-tau variants) is often thought to be a measure of all forms of tau phosphorylated at this epitope in CSF. However, this may not be correct. The clinically approved CSF p-tau181 tests (from commercial vendors including Elecsys®, Innotest® and Lumipulse®) target mid-region forms of tau phosphorylated at threonine 181 [120–122]. These assays follow the principle of sandwich ELISA: a capture antibody specific to the threonine-181 phosphorylation site partnered with an antibody that binds non-phosphorylated tau at the mid-region of the protein. This means that the CSF p-tau181 assays measure phosphorylated forms of tau metabolized from the soluble pool of brain-derived tau that contain the mid-region part of the protein. We now know that CSF contains both N-terminal and mid-region tau in highly measurable quantities [59, 123]. In comparison, C-terminal tau forms are poorly secreted into CSF [124, 125]. On the other hand, evidence has emerged that blood may be predominately consisting of N-terminal to mid-region forms of tau. Consequently, the development of p-tau assays for blood has focused on these species. Tatebe et al. [76] developed a p-tau181 assay by substituting the detection antibody in the Simoa™ Tau 2.0 total tau kit for a p-tau181-specific monoclonal antibody. Karikari et al. [79, 93] developed novel p-tau181 and p-tau217 assays targeting p-tau forms that also contain the N-terminal amino acid 6–18 epitope. Similarly, the Eli Lilly-developed p-tau181 and p-tau217 are located N-terminally to the p-tau sites [56]. Although each of the assays was primarily developed for use in blood, they are equally suitable for use in CSF [75, 92, 93]. By contrast, a p-tau assay that uses the same phosphorylation-specific antibody for both capture and detection (IMR method) thus exclusively targeting phosphorylation at threonine-181 irrespective of the fragment(s) on which this occurs [126] is increased not only in AD but also in other neurogenerative disorders [89]. Plasma Aβ42, Aβ40 and other Aβ peptides are proteolytic products of the amyloid precursor protein [127]. Plasma Aβ peptides are thought to be metabolic by-products of brain-derived extracellular amyloid plaques, although considerable peripheral sources of Aβ have been reported thereby reducing the specificity of plasma Aβ measures as biomarkers for brain pathophysiology compared to CSF Aβ [128]. Plasma Aβ assays include ELISA-based immunoassays on the Simoa [64], Elecsys [85] and IMR [129] platforms as well as IPMS assays [65, 66, 86, 130]. The IPMS assays involve enrichment of Aβ in plasma by immunoprecipitating with Aβ antibodies, either mid-region [66, 87] or N-terminal [65] coated onto paramagnetic beads. Stable isotope-labelled synthetic peptides corresponding to each Aβ peptide of interest (e.g. Aβ42, Aβ40 and Aβ38) are used as mass spectrometry quantification standards. However, in the case of the Nakamura et al. [87], Aβ38 is used as a single standard for all Aβ forms of interest.

This review recognizes some important limitations. The rating of phases and aims are, in some instances, based on limited studies from the same research group(s), which may introduce bias into our conclusions without independent findings. The online supplement clearly stipulates the studies evaluated in this review to provide clarity for the reader, however. In addition, the diagnosis of AD and MCI varied across studies (AD dementia; in vivo PET; neuropathological) as the standard of truth (e.g. post-mortem diagnosis) could not be applied to all studies. However, given the nature of current blood biomarker research, the majority of studies had at least one measure of cerebral Aβ pathology by either CSF or PET. Further, given the known association between CSF/PET neuropathological findings at post-mortem [131–133], we feel this is an adequate and more feasible assessment of than post-mortem diagnosis, particularly since this review focused on AD at the prodromal (i.e. MCI) stage where the interval between blood and post-mortem assessment would be considerable. We also recognize at the time of the review recent data on plasma p-tau231 could be included in the evaluation [83] despite being sporadically mentioned. A further limitation is that while we aimed to be as inclusive as possible when reviewing the literature, our search was not conducted as a formal systematic review. A number of PubMed research strings were proposed by the Biomarker Roadmap Initiative and adapted for individual projects; however, the literature databases and some selection criteria for included papers were chosen by the authors of each review, who could add papers from personal knowledge (e.g. preprints). Finally, in this review, we have assumed the same context of use adopted from 2017 by the Strategic Biomarker Roadmap, e.g. diagnosis in specialistic centres. However, the potential and the specific features of plasma biomarkers may require to validate and assess them considering screening and case finding as the proper contexts of use.

## Conclusion

In this review, we have assessed that stage of maturity of blood AD biomarkers (p-tau and Aβ) in context of the Biomarker Roadmap Initiative. Owing to the experience on fluid assessment built with CSF biomarkers, much has been achieved in a short period of time. Full achievements for p-tau have been obtained in phases 1 to 3, with partial achievement for Aβ. Plasma p-tau (p-tau217 and p-tau181) highlights AD in dementia cases with high accuracy and is validated by neuropathological studies. Plasma p-tau also can highlight future AD risk, which is comparable to CSF p-tau181 risk models. However, the field of blood biomarkers is undoubtedly lacking assay platform comparisons, clear preanalytical guidance and the effect of common covariates on biomarker levels – which will inform on accurate biomarker cut-offs. More information is needed on these secondary aims in phases 2 and 3 before phase 4 (real-world performance) and phase 5 (assessment of impact/cost) studies can be initiated for blood biomarkers.

## Data Availability

https://drive.switch.ch/index.php/s/4reUTSuqNZHyIC8
